# Skeletal Dysplasia Mutations Effect on Human Filamins’ Structure and Mechanosensing

**DOI:** 10.1038/s41598-017-04441-x

**Published:** 2017-06-26

**Authors:** Jonne Seppälä, Rafael C. Bernardi, Tatu J. K. Haataja, Maarit Hellman, Olli T. Pentikäinen, Klaus Schulten, Perttu Permi, Jari Ylänne, Ulla Pentikäinen

**Affiliations:** 1 Department of Biological and Environmental Science and Nanoscience Center, University of Jyvaskyla, P.O Box 35, Survontie 9 C, FI-40014 Jyvaskyla, Finland; 2 Beckman Institute for Advanced Science and Technology, University of Illinois at Urbana-Champaign, Champaign, 61801 USA; 3 Department of Physics, University of Illinois at Urbana-Champaign, Champaign, 61801 USA; 4 Department of Chemistry, University of Jyvaskyla, P.O Box 35, Survontie 9 C, FI-40014 Jyvaskyla, Finland

## Abstract

Cells’ ability to sense mechanical cues in their environment is crucial for fundamental cellular processes, leading defects in mechanosensing to be linked to many diseases. The actin cross-linking protein Filamin has an important role in the conversion of mechanical forces into biochemical signals. Here, we reveal how mutations in Filamin genes known to cause Larsen syndrome and Frontometaphyseal dysplasia can affect the structure and therefore function of Filamin domains 16 and 17. Employing X-ray crystallography, the structure of these domains was first solved for the human Filamin B. The interaction seen between domains 16 and 17 is broken by shear force as revealed by steered molecular dynamics simulations. The effects of skeletal dysplasia associated mutations of the structure and mechanosensing properties of Filamin were studied by combining various experimental and theoretical techniques. The results showed that Larsen syndrome associated mutations destabilize or even unfold domain 17. Interestingly, those Filamin functions that are mediated via domain 17 interactions with other proteins are not necessarily affected as strongly interacting peptide binding to mutated domain 17 induces at least partial domain folding. Mutation associated to Frontometaphyseal dysplasia, in turn, transforms 16–17 fragment from compact to an elongated form destroying the force-regulated domain pair.

## Introduction

Cells explore their environment by sensing and responding to mechanical forces^1, 2^. Fundamental cellular processes, such as cell migration, differentiation, and homeostasis, take advantage of this sensing mechanism^3^. At molecular level mechanosensing is mainly driven by mechanically active proteins. These proteins are able to sense and respond to forces by, e.g., undergoing conformational changes^3, 4^, exposing cryptic binding sites^5, 6^, or even by becoming more tightly bound to one another^7^. Defective responses to forces are known to cause a plethora of pathological conditions^8–11^, including cardiac failure^12^, as well as pulmonary injury^13^ and are also linked to cancer^14^.

In cell tissues, the connection between the actin cytoskeleton and the extracellular matrix enables the transmission of forces over long distances. Linking actin to extracellular matrix, Filamins (FLN) can simultaneously bind actin and the cytoplasmic domains of transmembrane receptor integrins. FLNs have been shown to be a central mechanotransduction element of the cytoskeleton^15^ and FLNs binding to integrins was shown to be force regulated^6, 16^. Besides actin and integrins, FLNs also bind and modulate over 90 other cellular proteins^17, 18^. FLNs are key players in the regulation of various processes in cells, including cell motility and signaling^17, 18^.

**Figure 1:**
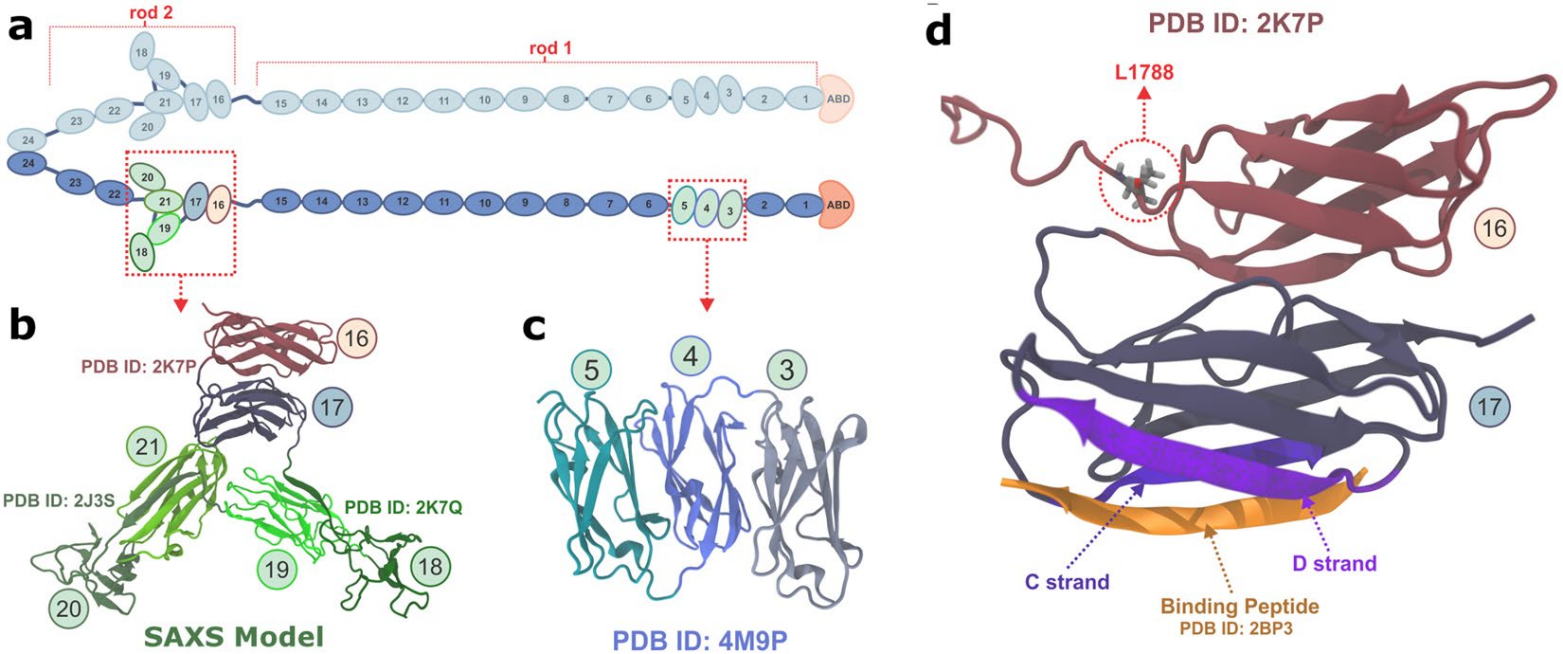
Schematic representation of Filamin. (**a**) Schematic representation of FLN dimer having 24 Ig-domains (blue) and the actin-binding domain (ABD, orange) in monomer. The two monomers are dimerized via domains 24. The atomic detailed structures of Ig-domains that form compact structures by interacting with the neighboring domains are highlighted and shown in green, and the domains 16 and 17 studied here are shown in light red and light blue, respectively. The structures of compact domain fragments are shown in panel (b) (FLNa16-21^21^) and in panel (c) (FLNa3-5^20^). The FLNa16-21 model^21^ shown in (**b**) is obtained from SAXS using the high resolution structures of FLNa16-17^22^, FLNa18-19^22^, and FLNa20-21^23^. (**d**) Shows the structure of the domain pair FLNa16-17^22^ studied here. The binding mode of GPIbα-peptide (orange) to groove between the strands C and D of FLNa17 is shown. The FLNa16-17 - GPIbα-peptide structure is obtained by superimposing FLNa17-GPIbα-peptide X-ray structure^26^ with FLNa16-17 structure^22^. L1788, whose mutation to arginine causes FMD^29^ is shown as ticks.

Three FLN coding genes are found in humans: X-chromosomal *FLNA*, and autosomal *FLNB* and *FLNC*. These genes encode their respective proteins FLNa, FLNb, and FLNc, all of them with high sequence similarity. FLNa is the most abundant isoform, whereas FLNb is usually expressed at low levels, and FLNc is mainly expressed in striated and cardiac muscle tissues^17, 18^. As shown in Fig. 1a, FLNs form a homodimeric structure (dimerization occurs at domain 24)^17, 18^, presenting an amino terminal actin-binding domain (ABD), followed by a chain of 24 immunoglobulin (Ig)-like domains. Two flexible hinge regions connect Ig-domains 15 to 16, and 23 to 24. These hinge regions divide Ig-domains into rod 1 (domains 1–15) and rod 2 (domains 16–23) (Fig. 1a).

Significant structural differences are found between rod 1 and 2, with the latter being more compact than the former^19^. Differences are partially explained by the fact that only three domains of total 15 rod 1 domains are known to form a compact multidomain fragment (domains 3–5)^20^ (Fig. 1a,c), whereas six of eight rod 2 domains form a compact propeller-like structure (domains 16–21)^21^ (Fig. 1a,b). In the 16–21 segment, domains 16–17^22^ form an interacting globular domain pair, while domains 18–19^22^ and 20–21^23^ form intertwined pairs (Fig. 1b). In both 18–19 and 20–21 domain pairs, the first beta-strands of even-numbered domains are folded with odd-numbered domains^22, 23^ (Fig. 1b). Also within rod 2, domain 21 is known as a hot spot binding site in FLNs, as several proteins are known to bind to this site, including transmembrane receptor integrins^24^ and glycoprotein Ib-IX-Vα (GPIbα)^25, 26^. Interestingly, the integrin and GPIbα binding sites in FLN19 and 21 are masked by the first β-strands of domains 18 and 20^22, 23^. These masked binding sites are revealed when shear-force is applied to FLNs^6, 16^. Hence, interacting FLN domains 18–19 and 20–21 function as mechanosensors. The function of other interacting domain fragments 16–17^22^ or 3–5^20^ has been remained enigmatic.

Various mutations, small deletions or insertions, truncating nonsense mutations and missense mutations have been characterized in all three FLN encoding genes, *FLNA, FLNB*, and *FLNC* ^27^. Mutations in FLNs are linked to a diverse array of congenital disorders influencing the development of central nervous system, cardiovascular system, muscle and connective tissue^18, 27^. The mutations span throughout the proteins, but disease-specific clustering of mutations are observed^27–32^. For instance, the majority of the missense mutations causing skeletal dysplasia’s, including otopalatodigitial spectrum disorders (OPDSDs) and Larsen syndrome-Atelosteogenesis (LS-AO) spectrum, locate at ABD and domains around the hinge region between FLN rods 1 and 2^27–32^. These two syndromes caused by mutations in FLNa (OPDSDs) and FLNb (LS-AO), share phenotypic similarities causing defects in joint and limb bone formation and facial abnormalities. The mutations causing both OPDSDs and LS-AO disorders are classified as gain-of-function mutations, while other clinically distinct disorders, such as X-linked Periventricular Nodular Heterotopia (OMIM #300049), are caused by loss-of-function mutations^27^. It is however not yet understood how some missense mutations in FLNa and FLNb lead to OPDSDs and LS-AO among other skeletal disorders. It has been reported that OPDSD-associated mutations in ABD can enhance acting binding, suggesting that they might be caused by disruption of the mechanosensory properties of FLN^32, 33^.

Here, we have studied how the skeletal dysplasia-associated missense mutations at FLN Ig-domains 16–17 affect structures and functions of these domains. The studied mutations were L1788R in FLNa16, and G1834R and S1902R in FLNb17. The FLNa16’s L1788R mutation is linked to the frontometaphyseal dysplasia (FMD, OMIM #305620), which belongs to OPDSD family^29^. FMD is characterized by hyperostosis of the skull and modeling anomalies of the tubular bones as well as tracheobronchial cardiac and urological malformations^29^. The two FLNb17 mutations, G1834R and S1902R, are LS-associated mutations (OMIM #150250)^31, 32^. LS features joint dislocations and malformations of the cervical spine as well as supernumerary carpal and tarsal ossification centers^32^. Both FMD and LS are dominantly inherited disorders.

Domains 16–17 form a unique compact domain pair in Ig-domain chain in FLN, as shown in Fig. 1d. The GPIbα adhesion receptor binds in a groove between the β-strands C and D of domain 17 by forming an additional β-strand^26^, as shown in Fig. 1d. Otherwise the role of 16–17 fragment in FLN is not understood. Based on the high resolution structure of FLNa16-17^22^, the L1788R mutation is located close to the interface between domains 16 and 17, as shown in Fig. 1d. The structure of corresponding domains in FLNb were not known, however based on 69% sequence identity, the structure was believed to be very similar to the one of FLNa.

Here, we present the crystallographically-resolved atomic structure of domains 16 and 17 of the human FLNb, and also how skeletal dysplasia associated mutations interfere in the structure and function of both FLNa and FLNb. Combining state-of-the-art experimental and simulation techniques is crucial to resolve the structure and function of large macromolecular complexes such as FLNs^34, 35^. Employing several experimental structural biology techniques and molecular dynamics (MD) simulations, our mutational studies show that FMD associated mutation on FLNa16 change the structure of FLNa16-17 from compact and rigid to elongated and flexible. The two FLNb17 mutations associated with LS both destabilize the internal structure of domain 17, and S1902R mutation almost completely unfolds it. Interestingly these mutations do not abolish the binding of a model peptide GPIbα to FLNb17, working as a prototype for many possible interaction partners of domain 17^36^ (Fig. 1d). The peptide binding was also observed to induce FLNb17 S1902R refolding. LS-linked mutations were also observed to increase FLN’s susceptibility for proteolytic digestion. Our results also show that shear forces are able to break the interaction between domains 16 and 17, for both FLNa and FLNb, with the aforementioned mutations reducing the necessary force to break the inter-domain interaction or domain folding. Accordingly, the molecular mechanics underlying FMD and LS are likely related to mechanoregulation of mutated FLN16-17, but especially with LS, decreased expression level of mutant proteins due to their rapid degradation in cells might also be also attributed to the pathogenesis of the diseases. Taken together, our results provide new information on FLN’s mechanosensory hotspots, and also shed light over the molecular mechanism of skeletal dysplasias.

## Results

### The structure of FLNb16-17

**Figure 2:**
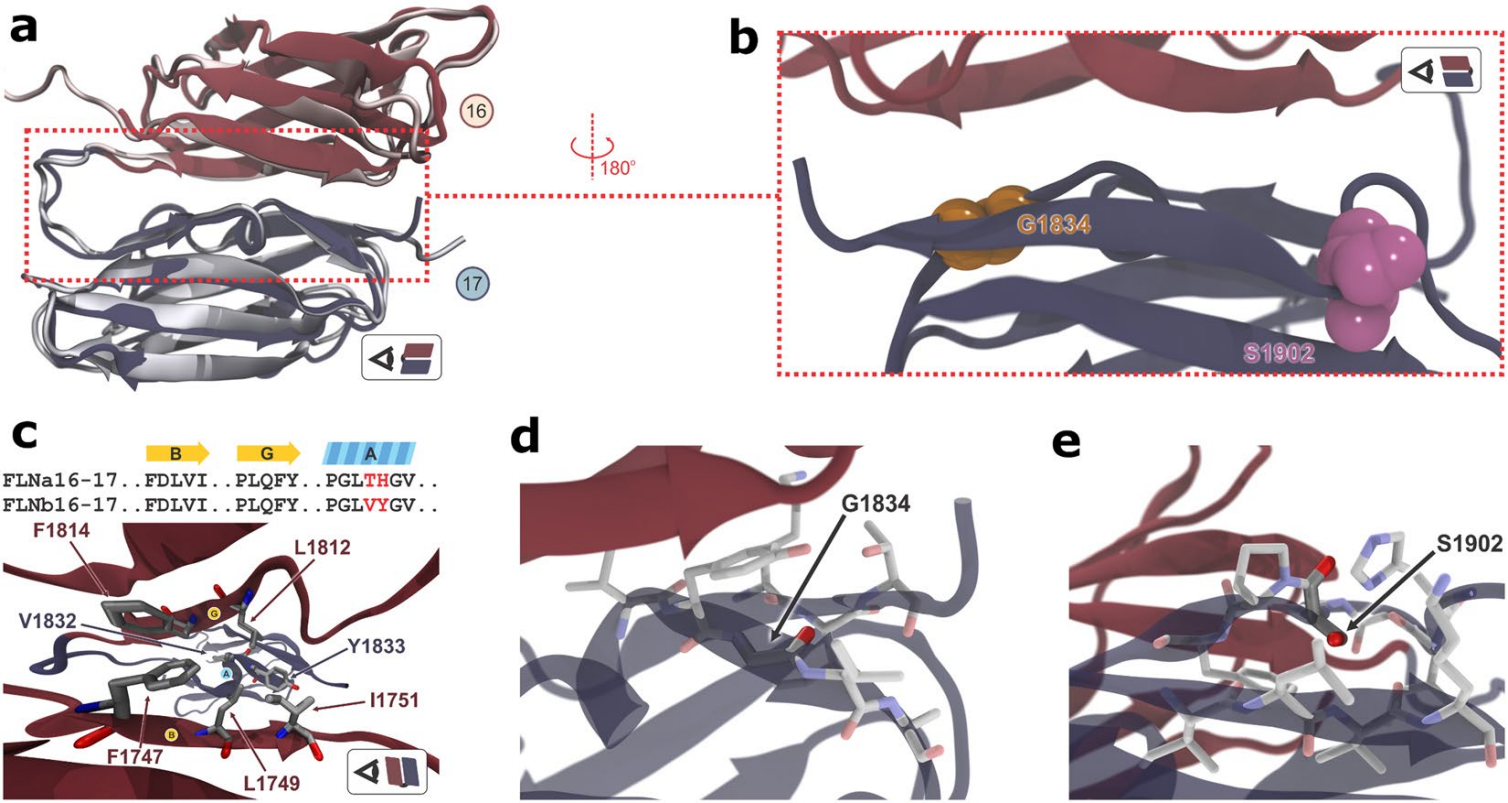
Crystal structure of FLNb16-17. (**a**) The crystal structure of FLNb16-17 (FLNb16 dark red and FLNb 17 dark blue) superimposed with FLNa16-17^22^ (grey). (**b**) A zoomed view to the interface of domains 16 and 17. G1834 and S1902, whose mutation to arginine causes LS^31, 32^, are shown as space filling model (G1834 with orange and S1902 with magenta). (**c**) The sequence alignment of residues at the inter-domain interface of FLNa16-17 and FLNb16-17. The residues that are not conserved are shown in red in the alignments. The key residues forming the interaction in FLNb16-17 are shown as ball-and-sticks. (**d**,**e**) The closer view to the locations of amino acids whose mutations to arginine cause LS. Both G1834 (**d**) and S1902 (**e**) in FLNb17 (shown in ball-and-stick) are buried. Coloring of domains are similar as in panel (a).

To characterize the possible mechanism of mechanosensing attributed to FLNb and the role of its domain 17 mutations (G1834R and S1902R) in LS we need first to unravel the atomic structure of the human FLNb16-17. The two-domain fragment of wild type (WT) human FLNb16-17 was expressed, purified and crystallized. The obtained crystals belonged to space group P321 and diffraction data up to 2.5 Å resolution were used (Table 1). The asymmetric unit contained two copies of FLNb16-17 (Supplementary Figure 1). The structure was solved by molecular replacement using FLNa16-17 (the protein data bank (PDB) ID 2K7P)^22^ as a search model. The electron density of following amino acids were not seen and thus not included in the final model; chain A 1739–1744, 1849–1852 and chain B 1739–1744, 1820, 1821, 1847–1851, 1872, 1873. In the final model, the two FLNb16-17 molecules were nearly identical (the root mean square deviation, RMSD 0.16 Å). The structure of FLNb16-17 (Fig. 2a) was observed to be highly similar to the one of FLNa16-17, with RMSD of 0.96 Å (145 Cα atoms) when all 40 NMR structures of FLNa16-17 were used in the RMSD calculation (RMSD of 40 NMR structures itself is 0.5 Å). FLNb16-17 structure was deposited in with PDB ID 5DCP.

Key residues responsible for the interdomain interactions in both FLNa16-17 and FLNb16-17 are found conserved, with the exception of T1876 and H1877 in FLNa17, which are substituted by V1832 and Y1833 in FLNb17 (Fig. 2c). These substitutions have only minor influence on the domain-domain interactions; the V1832 in FLNb17 cannot form a hydrogen bond with FLNb16’s Q1813 as T1876 in FLNa17 forms with Q1857. Another difference is at edge of domain-domain interface, R1789 in FLNa16 is substituted by K1745 in FLNb16. While R1789 in the isoform “a” points inwards in most of the NMR-structures forming interdomain hydrogen bonds, K1745 in the isoform “b” points outwards in the crystal structure. The amino acid residues G1834 and S1902, whose mutations to arginine cause LS^31, 32^, are located close to the domain-domain interface at β-strands A and G, respectively (Fig. 2b). The Cα-atom of G1834 points inwards of Ig-domain 17 (Fig. 2d). Also the side chain of S1902 is not present at the Ig-domain 17 surface, but instead it is masked by nearby amino acid side chains (Fig. 2e), especially those from P1903 and H1898. Both LS-linked G1834 and S1902 residues are totally conserved among vertebrates as seen from the FLN vertebrate sequence alignment shown in Supplementary Figure 2. Accordingly, the mutations of residues G1834 and S1902 to arginine likely influence the local domain architecture.

### The filamin-A and the FMD-causing L1788R mutation

**Figure 3:**
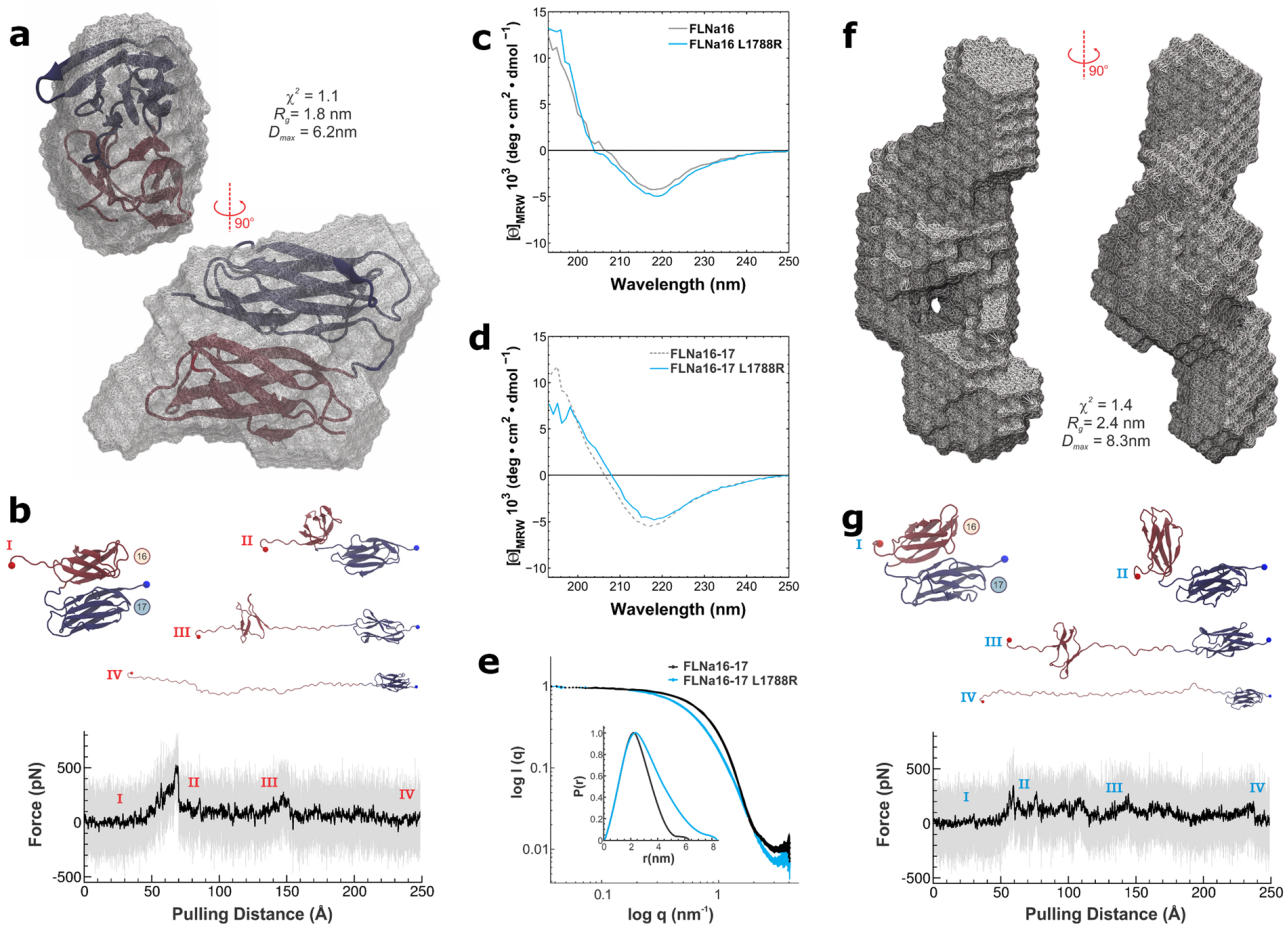
Effects of FMD associated mutation on the structure of FLNa16-17. (**a**) The *ab initio* shape envelopes calculated form SAXS data shows that FLNa16-17 WT is globular in accordance with the NMR-structure of FLNa16-17^22^, which is superimposed with the SAXS envelope. The molecular dimensions in terms of *R*_*g*_ (radius of gyration) and *D*_*max*_ (maximum dimensions), as well as, the fit of the shape envelope on the experimental scattering curve (χ^2^) are shown. (**b**) Unfolding trace of FLNa16-17 WT obtained from SMD a pulling speed of 2.5 Å ns^−1^ shows that interaction between domains 16 and 17 is lost first, followed by domain 16 unfolding. The full trajectory is shown gray. The black line represents a moving average with a box size of 500 steps. The snapshots of different time steps are labelled I-IV. (**c**,**d**). The CD-spectroscopy analyses of the WT and mutated FLNa16 (**c**) and FLNa16-17 (**d**) shows that L1788R does not destroy the β-sheet folding of FLNa16. (**e**,**f**). The experimental scattering data, the distance-distribution function, *P(r)*, (insert), and the *ab initio* shape envelope obtained from SAXS measurements show that WT FLNa16-17 (panel A) is globular but L1788R mutated FLNa16-17 is elongated (**f**). The scattering data shown in **e** is scaled to the same forward scattering intensity *I(0)*. The molecular dimensions of the L1788R envelope in terms of *R*_*g*_ and *D*_*max*_, as well as, (χ^2^) are shown in (**f**). (**g**) The unfolding trace of FLNa16-17 L1788R obtained from SMD a pulling speed of 2.5 Å ns^−1^ shows that similarly As with WT, the interaction between domains 16 and 17 is lost first, followed by domain 16 unfolding. However, the force needed to unfold L1788R mutated FLNa16-17 is notably lower than that needed for WT. The full trajectory is shown gray. The black line represents a moving average with a box size of 500 steps. The snapshots of different time steps are labelled I-IV.

Domains 16 and 17 of FLNa exhibit a globular compact fold, as shown in Fig. 3a by FLNa16-17’s structure resolved by NMR (PDB ID 2K7P)^22^ and by the envelope modelled based on our small-angle X-ray scattering (SAXS) data. L1788, whose mutation to arginine is linked to FMD is highly conserved in vertebrate FLNs as seen from the sequence alignment shown in Supplementary Figure 2. The influence of L1788R mutation on FLNa16-17 structure was studied combining various biophysical, biochemical and structural techniques. Circular Dichroism (CD) spectroscopy was employed to study the effects of L1788R mutation on FLNa16 and FLNa16-17 folding. The CD spectra of the WT FLNa16 had a minimum at 218 nm and a conversion to positive ellipticity at 220 nm (Fig. 3c **)** corresponding to high proportion of β-strands, consistent with the detailed atomic structure^22^. FLNa16 L1788R had a very similar CD spectrum to FLNa16 WT, suggesting that this mutation does not have major effects on FLNa16 folding (Fig. 3c **)**. The CD spectra of the two domain FLN fragments, FLNa16-17 WT and L1788R, were found to be highly similar to those of single domains (Fig. 3d).

The fluorescence-based thermal stability assay, which is based on the binding of hydrophobic dye to protein, and limited proteolysis were further used to investigate the effects of L1788R mutation on FLNa16-17 structure. FLNa16-17 L1788R shows similar melting curve to FLNa16-17 WT, albeit with slightly decreased thermal stability compared to the WT fragment **(**Supplementary Figure 3a **)**. The results from limited proteolysis showed that FLNa16-17 L1788R was more susceptible to chymotrypsin digestion than WT FLNa16-17 (Supplementary Figure 4 **)**. After 180 min, only very little of FLNa16-17 L1788R was left whereas FLNa16-17 WT is almost completely intact, with similar amount of protein at the beginning and the end of the chymotrypsin treatment.

The effect of the L1788R mutation on the overall structure of FLNa16-17 was studied using SAXS. The scattering curves, distance distribution functions, and *ab initio* shape envelopes show that this mutated FLNa16-17 is more elongated than the WT fragment (Fig. 3e,f and Supplementary Table 1 **)**. Furthermore, the normalized Kratky profile^37^ showed that FLNa16-17 L1788R is more flexible than WT FLNa16-17 **(**Supplementary Figure 5 **)**. Taken together, it seems that L1788R mutation does break the tight interaction between domains 16 and 17 but does not cause major changes on domain 16 folding.

To investigate how domains 16 and 17 of FLNa respond to shear force we carried out steered molecular dynamics (SMD)^38^ simulations, implemented in NAMD^39^. SMD was used mainly to characterize how stable the FLNa16-17 globular shape is for both WT and L1788R mutant proteins. The stability of the FLNa16-17 structure (PDB ID 2K7P), and of the domains 16–17 compact complex, was investigated employing a similar protocol described in the investigation of ultrastable protein complexes^7^. The L1788R mutant was prepared in silico in VMD^40^, employing the FLNa16-17 WT structure, and following QwikMD^41^ protocols. FLNa16-17 was pulled from its C-terminus at a pulling speed of 2.5 Å/ns, whereas the position of its N-terminal was restrained. The applied external force first induced the opening of the compact domain-domain arrangement in both FLNa16-17 WT (Fig. 3b) and FLNa16-17 L1788R mutant (Fig. 3g). As shown by the plots’ first peak (Fig. 3b and g), the necessary force to break the complex open was found to be significantly lower for the mutant protein, averaging about 300pN compared to over 550pN for the WT protein. After the domain-domain interface is exposed, the folding of domain 16 is unraveled with similar force traces presented for both WT and mutant proteins. The folding of domain 17 stayed intact during the whole SMD simulation.

### The filamin-B and the LS-associated mutations

**Figure 4:**
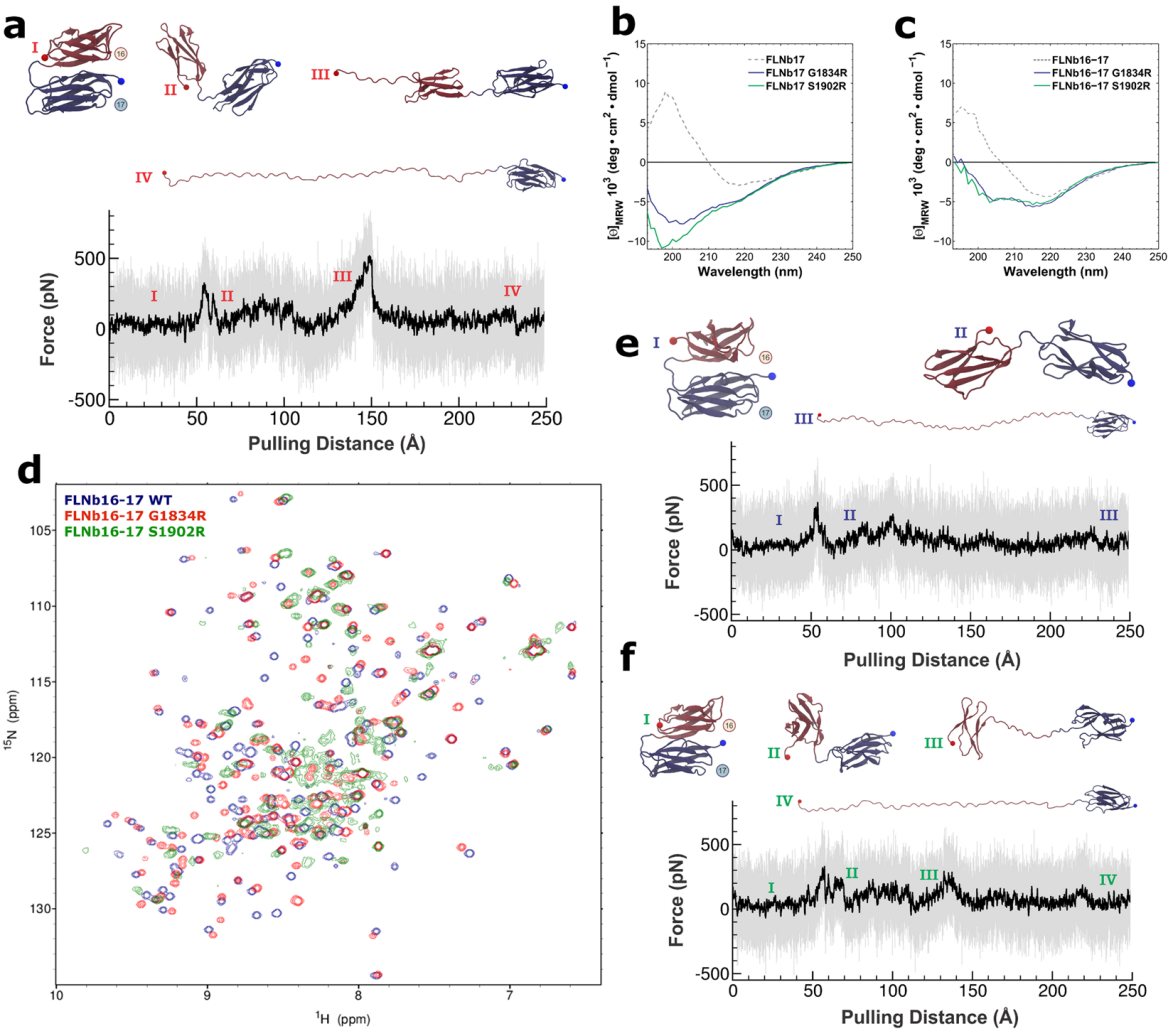
Effects of G1834R and S1902R mutations on FLNb16-17 structure and force response. (**a**) Unfolding trace of FLNb16-17 WT obtained from SMD a pulling speed of 2.5 Å ns^−1^ shows that the interaction between domains 16 and 17 is lost first, followed by domain 16 unfolding. The full trajectory is shown gray. The black line represents a moving average with a box size of 500 steps. The snapshots of different time steps are labelled I-IV. (**b**,**c**) The CD-spectroscopy analyses of the WT and mutated FLNb17 (**b**) and FLNb16-17 (**c**) shows that both G1834R and S1902R destroy the β-sheet folding of FLNb17 and the neighboring domain 16 do not stabilize the β-sheet folding of mutated FLNb17 (**c**). (**d**) ^1^H ^15^N HSQC spectra of FLNb16-17 WT, G1834R and S1902R show that S1902R has high proportion of unfolded regions, whereas G1834R is mainly structurally ordered having only some disordered regions. (**e**,**f**) Unfolding traces of FLNb16-17 G1834R (**e**) and S1902R (**f**) obtained from SMD a pulling speed of 2.5 Å ns^−1^. Similarly as with FLNb16-17 WT (**a)**, the interaction between domains 16 and 17 is lost before domain 16 unfolding. However, with both mutants, forces needed to unfold domain 16 are considerable lower than seen with WT. The snapshots of different time steps are labelled I-IV.

The new structure of domains 16 and 17 of FLNb was also studied employing SMD. Curiously, unlike seen with FLNa16-17, with FLNb16-17 the necessary force to break the interaction between domains 16 and 17 is smaller than the force necessary to unfold domain 16, as shown in Fig. 4a. The necessary average force to break the WT FLNb16-17 complex open was a little over 300pN, significantly lower than the 500 pN force necessary to break open the WT FLNa16-17 shown in Fig. 3b. However, even though domain 16 was still the only one to unfold in our simulations, the required force to start unfolding this FLNb domain was found to be much higher than in the case of the same domain in FLNa (Figs 3b and 4a).

The CD spectra of the WT FLNb17 is very similar to that of FLNa16, corresponding β-sheet folded structure (Figs 3c and 4b), and consistent with the atomic structure of FLNb16-17 (Fig. 2). Interestingly, the LS associated mutations in FLNb17 (G1834R and S1902R) were found to significantly change the folding of the FLNb17 (Fig. 4b). Both FLNb17 mutants displayed a minimum around 200 nm, which is a characteristic sign of unfolded or disordered structures (Fig. 4b). S1902R mutant has a deeper minimum around 200 nm than S1834R indicating higher proportion of unfolded structure in FLNb16-17 S1902R than in G1834R mutant. Unfolded region is also seen in the CD spectra of G1834R or S1902R mutated FLNb16-17 (Fig. 4c). Accordingly, the neighboring domain 16 does not seem to provide significant stabilization nor induce folding of mutated FLNb17.

The fluorescence-based thermal stability assays supported the assumption of a disordered structure for G1834R and S1902R mutated FLNb17. FLNb16-17 WT showed clear melting transition, as expected but the G1834R and S1902R mutated FLNb16-17 fragments showed nearly or completely featureless decaying curves, respectively, which are typical for un- or misfolded proteins^42^ **(**Supplementary Figure 3b **)**. The limited protease digestion showed that FLNb16-17 G1834R and S1902R were significantly more readily digested by chymotrypsin than WT FLNb16-17 suggesting that unfolded regions are present in FLNb16-17 **(**Supplementary Figure 6 **)**. At the 60 min time point the WT FLNb16-17 was largely intact, whereas no full-length species were left for mutant proteins. The fragmentation of the FLNb S1902R mutant is faster than that of G1834R mutant, and both LS-linked mutants are digested significantly faster than seen for FLNa16-17 L1788R.

In order to get more information about structural changes caused by LS associated mutations (G1834R and S1902R), ^1^H, ^15^N heteronuclear single quantum coherence (^1^H ^15^N HSQC) NMR spectra were recorded for FLNb16-17 WT, G1834R and S1902R fragments. Two domain fragments were used, as we were not able to express enough ^15^N-labelled single domain fragments FLNb17 G1834R and S1902R for NMR measurements. The ^1^H ^15^N HSQC spectrum of FLNb16-17 S1902R clearly shows that S → R mutation disrupted structural integrity of this fragment as many of FLNb16-17 S1902R ^1^H, ^15^N cross peaks are exchange broadened and cluster in the central region of the spectrum (Fig. 4d). On the contrary, based on ^1^H ^15^N HSQC spectrum, the structural integrity of G1834R mutant sustains much better in comparison to S1902R. However, G1834R mutation clearly affects the structure of FLNb16-17 as can be inferred by comparing overlaid FLNb16-17 WT and G1834R ^1^H ^15^N HSQC spectra (Fig. 4d). Indeed, most of the FLNb16-17 WT and G1834R cross peaks cannot be superimposed and some of G1834R cross peaks cluster in the middle of ^1^H chemical shift range and display exchange broadening, indicative of partial collapse of the structure.

Taken together, combining our results from CD spectroscopy, thermal stability, limited proteolysis assays, and NMR spectroscopy suggest that S1902R mutated FLNb17 has high proportion of unfolded regions in both isolated FLNb17 and in the context of two domain fragment FLNb16-17. G1834R mutation also clearly destabilizes the structure of FLNb17 but FLNb16-17 G1834R is mostly folded.

SMD simulations of mutants showed no major change in the necessary force and steps to break domain-domain interface in the WT (Fig. 4a), G1834R mutant (Fig. 4e), and S1902R mutant (Fig. 4f). Interestingly, both G1834R and S1902R mutations in the domain 17 affect the unfolding of domain 16. As seen from Fig. 4a,e and f, in both G1834R and S1902R mutated FLNb16-17 fragments lower forces are needed to unfold domain 16 than in the FLNb16-17 WT fragment. G1834R or S1902R mutated domain 17 continues stable in the whole simulation, as shown in Fig. 4e and f.

**Figure 5:**
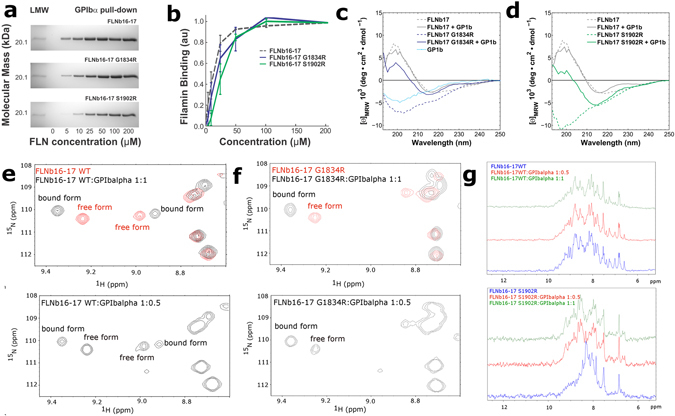
Effects of G1834R and S1902R mutations on FLNb16-17 interactions. (**a**,**b**) The binding assays show that FLNb16-17 G1834R and S1902R mutants are able to bind the GP1bα-peptide almost similar avidity as FLNb16-17 WT despite of the fact that these mutation unfold domain 17. GP1bα-peptide binding to FLNb16-17 WT and G1834R and S1902R mutants in 5, 10, 25, 50, 100, and 200 μM concentrations is shown. FLNb16-17 WT and G1834R and S1902R mutants binding to GP1bα-peptide was quantified by protein staining and expressed as FLN binding (in arbitrary units) calculated as the ratio of FLN bound to FLN in the loading control, normalized to maximal FLN binding in each experiment (mean S.E. (error bars); n ≥ 3). (**c**,**d**) The CD-spectrometry measurements show that the GP1bα-peptide binding to G1834R (**c**) and S1902R (**d**) mutated FLNb17 induces β-sheet folding on these domains. (**e**,**f**) Selected regions of HSQC spectra of ^15^N-labelled FLNb16-17 WT and G1834R fragments collected before and after titration with GP1bα-peptide shows tight binding of the peptide to both WT (**e**) and G1834R (**f**) fragments. (**g**) One-dimensional ^15^N-edited, ^1^H spectra of FLNb16-17 WT and S1902R with increasing concentration of GPIbα peptide. Spectra for S1902R mutant highlight increasing dispersion of amide proton chemical shifts upon titration of GPIbα-peptide, indicative of growing structural order.

FLNs execute many of their functions *via* interactions with other proteins^18^. The GPIbα adhesion receptor binds in a groove between the β-strands C and D of domain 17 by forming an additional β-strand^26^ (Fig. 1d), and is a prototype of many possible interaction partners of domain 17^36^. Based on our aforementioned results, the LS-associated mutations of FLNb17 disrupt the domain folding, which suggests that FLNb17 interaction with a GPIbα-peptide would not be possible. Surprisingly, pull down experiments showed that G1834R and S1902R mutated FLNb16-17 interact with the GPIbα-peptide similarly to the WT fragment (Fig. 5a and b). This suggests that the peptide interaction with mutated FLNb17 induces β-sheet folding. To confirm this hypothesis, CD spectra of G1834R and S1902R mutated FLNb17 were recorded with the added GPIbα-peptide. The binding of the GPIbα-peptide to both the G1834R and S1902R mutated FLNb17 domain changed the minimum in the CD spectra from ~200 nm to 215 nm, indicating β-sheet formation (Fig. 5c and d). The CD spectra of mutated FLNb17 with the bound peptide were similar with WT FLNb17. The titration of ^15^N-labelled FLNb16-17 WT, G1834R and S1902R fragments with GPIbα-peptide further confirmed the binding of GPIbα-peptide to both WT and mutated proteins. The GPIbα-peptide binds to both WT and G1834R mutant as seen from ^1^H ^15^N HSQC spectra of WT and G1834R fragments shown in Supplementary Figure 7. At protein-GPIbα-peptide concentration ratio of 1:0.5, two sets of cross peaks for some residues can be observed, representing free and peptide bound forms of FLNb16-17 WT and G1834R (Fig. 5e and f). Hence, both WT and G1834R are in slow exchange with the GPIbα-peptide on the NMR timescale. We take this as an indication of slowly dissociating complex (K_d_ < 10^−6^ M) between protein and GPIbα-peptide for both FLNb16-17 WT and G1834R. The titration of FLNb16-17 S1902R mutant with the GPIbα-peptide induced partial folding of S1902R structure, which can be seen as sharper amide cross peaks at the chemical shift regions typical for folded proteins (Supplementary Figure 7). GPIbα-peptide binding induced folding of FLNb16-17 S1902R can readily be seen by comparing 1D ^15^N-edited ^1^H spectra of S1902R mutant before and after titration with different peptide concentrations. Indeed, appearance of amide proton resonances outside the random coil ^1^H chemical shift range (7.5–8.5 ppm) upon addition of the GPIbα-peptide indicates nascent S1902R structure (Fig. 5g). Regrettably, owing to small amounts of ^15^N-labelled S1902R mutant available, we were not able to determine binding kinetics reliably as no clear observation of cross peaks corresponding particular residues in their free and peptide bounds forms was made. However, our data suggest that the G1902R binding constant is on the similar range with G1834R given that after the nominal 1:0.5 protein:peptide concentration ratio, no further chemical shift perturbations were observed upon addition of GPIbα peptide in 1:1 and 1:2 ratios. In other words, the results from both CD spectroscopy and NMR show that although LS-associated mutations disrupt the folding FLNb17, the peptide binding at least partially folds it.

### Mechanosensing

Mechanically active proteins are of paramount importance as mechanical responses are fundamental for several cellular processes. At the molecular level, understanding how mechanical signals are transmitted through these proteins may lead us to a better understanding of amino-acid residues that are essential for the mechanosensing function. The mechanical signals are frequently transmitted as allosteric signals that can be investigated by using statistical mechanics approaches to analyze MD trajectories^43^. Analyzing cross-correlation of fluctuations of atoms positions in these SMD trajectories we can calculate how force propagates through FLNs domains. For an ultrastable protein complex^7^, it was recently reported^44^ that when force is propagated through a molecule, soft degrees of freedom are stretched out along the path of force propagation, while stiff degrees become more important for the dynamics of the system. Therefore, paths where the motion is highly correlated are describing the paths along which force propagates through a biomolecular system^44^.

**Figure 6:**
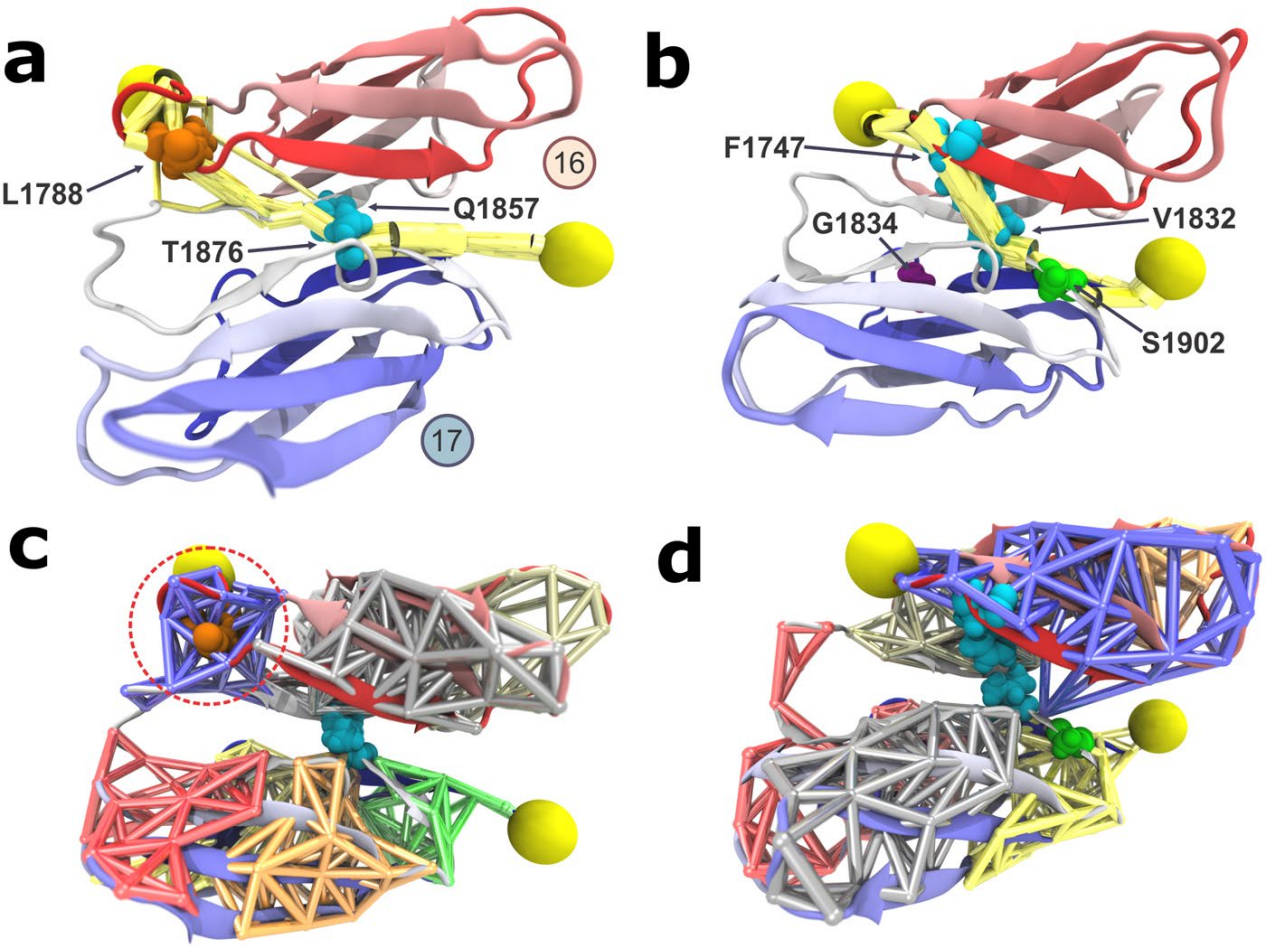
Network analysis and force propagation through FLN 16-17. When force is propagated through a molecule, soft degrees of freedom will be stretched out along the path of force propagation, while stiff degrees become more important for the dynamics of the system. Consequently, paths with high correlation of motion describe the paths along which force propagates through the system. (**a**) The calculated force propagation pathway shows that residue L1788, associated to FMD, is a main node in the force propagation pathway (yellow) in FLNa16-17. The analyses also revealed the strongest correlation between the internal motion of domains 16 and 17 occur between residues Q1857 and T1876. (**b**) The analyses revealed that in FLNb from LS associated residues G1834 is not part of the force propagation pathway (yellow) while S1902 contributes to the pathway but it is not a main node. (**c**) Community analysis reveals the highly correlated regions within the protein complex. It shows that L1788 is an important part of one of the network communities in FLNa (marked by the red dashed circle). (**d**) For FLNb, the mutation sites G1834 and S1902 are not shown as main nodes in the communities.

The force propagation pathway calculated for FLNa16-17 shows that residue L1788 is a main node in the force propagation network, as shown in Fig. 6a. Also, community analysis reveal that amino-acid residue L1788 is an important part of one of the network communities, which are obtained by applying the Girvan-Newman algorithm^45^, implemented in the dynamic network analysis tool^43^ of VMD^40^. Communities correspond to sets of residues that move in concert with each other^46^. As marked by the red dashed circle in Fig. 6c, L1788 is present as a main node of the community corresponding to the terminal region of FLNa16 and that also includes the short linker between domains 16 and 17. The significance of L1788 in FLNa’s function is supported by the fact that L1788 is conserved throughout vertebrates as seen from the sequence alignment shown in Supplementary Figure 2. For FLNb, the importance of the mutation sites for the force propagation pathway appears smaller, as shown in Fig. 6b. FLNb’s G1834 is not part of the force propagation pathway while S1902 contributes to the pathway but it is not a main node, with force propagation mostly bypassing it. Analyses of FLNb16-17 communities do not reveal any major contribution of the mutation sites, as illustrated in Fig. 6d.

The force propagation analysis also reveal that the strongest correlation between the internal motion of domains 16 and 17 occur between residues Q1857 and T1876 for FLNa and residues F1747 and V1832 for FLNb (Fig. 6a,b). These residues are conserved among vertebrates, either completely (FLN16 residues Q1857 and F1747) or alternating between structural analogs serine (T1876 in FLNa), and (V1832 in FLNb) (Supplementary Figure 2). Accordingly, Q1857 and T1876 in FLNa, and F1747 and V1832 in FLNb are likely representing an important connection between domains 16 and 17.

## Discussion

Mechano-regulated proteins control a myriad of cellular functions, e.g., cell migration and differentiation. As an important mechano-regulated protein complex, the actin crosslinking protein FLN has a crucial role in the conversion of mechanical cues into biochemical signals. Mutations in FLN are associated to many genetic disorders, including defects in brain, heart and skeleton^18, 27^. Molecular mechanisms behind these disorders have mainly remained unclear. Combining experimental and computational approaches, we present here the structure of FLNb domains 16 and 17, as well as an investigation on the mechanisms in which disease-associated mutations affect FLNa and FLNb mechanics.

Comparison of the NMR structure of FLNa16-17^22^ and the new X-ray structure of FLNb16-17 shows that, in their WT forms, both FLNa16-17 and FLNb16-17 have similar compact two-domain arrangement. As the function of this domain pair has so far remained enigmatic, we investigated how they respond on applied force. Our results from SMD simulations represent that when shear-forces are applied to FLN16-17, in both “a” and “b” isoforms, the domain-domain interface is broken first, followed by domain 16 unfolding. Domain 17 is not affected during simulations. In FLNb16-17 lower forces are required to break domain-domain interface than in FLNa16-17. Accordingly, the performed SMD simulations suggest that one possible function of the domain-domain interactions here is to stabilize domain 16. However, to this date, no data have been reported whether the domain-domain interface opening and/or domain unfolding happens also in living cells.

Deregulation of mechanosensory functions of FLN has been suggested to play key role in various filaminopathies. For example, OPDSDs, which are associated to ABD’s mutations and known to increase FLN’s affinity to F-actin, are linked to altered mechanosensory functions of FLN^27, 33^. It has also been suggested that other mutations in FLN’s Ig-domains affect the FLN’s mechanosensing functions, leading to phenotypes similar to those presented in the ABD’s mutations^47^. Furthermore, unfolding of FLNa23 with consequent weakening of the elasticity of FLNa/F-actin network under high mechanical stress has earlier been connected to FMD and Periventricular Nodular Heterotopia causing mutations^48^. As the structure of FLN16-17 is force regulated, it is not discounted that altered mechanosensory functions of FLN would be linked to both FMD and LS. Our results from SAXS and CD spectroscopy experiments clearly show that L1788R mutation in FLNa16 linked to FMD destroy the compact domain-domain arrangement transforming the mutated FLNa16-17 to elongated and flexible form, however, without affecting FLNa16 folding. Based on dynamical network analysis performed for trajectories obtained from SMD simulations, L1788 is a crucial amino acid residue in force propagation through FLNa16-17. Accordingly, these results suggest that altered mechanoregulation of FLNa16-17 might be linked to FMD caused by L1788R mutation.

With LS, the molecular mechanism seems to be more complicated. The results from CD spectroscopy, NMR, and fluorometric thermal analyses show that both G1834R and S1902R mutations destroy at least partially β-sheet folding of FLNb17 Ig-domain. This destroys the mechanoregulated, compact FLNb16-17 fragment. However, the dynamical network analysis performed for trajectories obtained from SMD simulations did not provide any evidence that G1834 and S1902 would be critical nodes in the force propagation pathways, but S1902 contributes to pathway. Accordingly, defects in mechanoregulation might have role in G1834R and S1902R mutations caused LS but alternative mechanisms may also exist. Based on our proteolysis experiments, one possible mechanism would be FLN’s increased susceptibly to proteolytic digestion in G1834R and S1902R mutations caused LS. However, since these mutants cause phenotypes in patients as heterozygotes, and since these phenotypes are different than those of null alleles^27^, we can speculate that either the degradation products of the mutants or the full length mutant polypeptide have specific deleterious functions in bone and cartilage tissue. Of note, at least one LS-AO mutant affecting FLNb domain 15 (P1699S) has been expressed as full length polypeptide in human embryonic kidney and carcinoma derived cell lines^31^.

FLNs’ execute many of their functions *via* interactions with other proteins. Accordingly, changes in interactions and interactions affinities might be linked to FLN associated disorders. Domain 17 has well characterized binding site for other proteins, and the binding mode of FLNa17- GPIbα-peptide has been resolved^26^. GPIbα-peptide binds in a groove between the C and D β strands, which is the conserved motif for FLN binding. Several other proteins have also been mapped to bind to domains 16 and 17^18, 49^. Our results show that LS-associated mutations cause FLNb17 misfold at least partially, but surprisingly, GPIbα-peptide binding to FLNb17 is not affected. This suggests that LS is not necessarily linked to changes in FLN’s interactions with other proteins. FMD causing L1788R mutation transforms the compact WT FLNa16-17 fragment to elongated form, simultaneously exposing new interfaces for interacting partner binding. Interaction between FLNa and FOXC1 transcriptor factor has been linked to pathogenesis of FLNa-linked skeletal disorders as the skeletal phenotypes caused by FOXC1 loss-of-function mutations and FLNA gain-of-function mutations resemble each other’s^50^. FOXC1 has been reported to bind to multiple domains in FLNa, including rod 1 domains 4-9 and rod 2 domains 16-21. However, the binding mode of FOXC1 to FLNa is currently not known.

**Figure 7:**
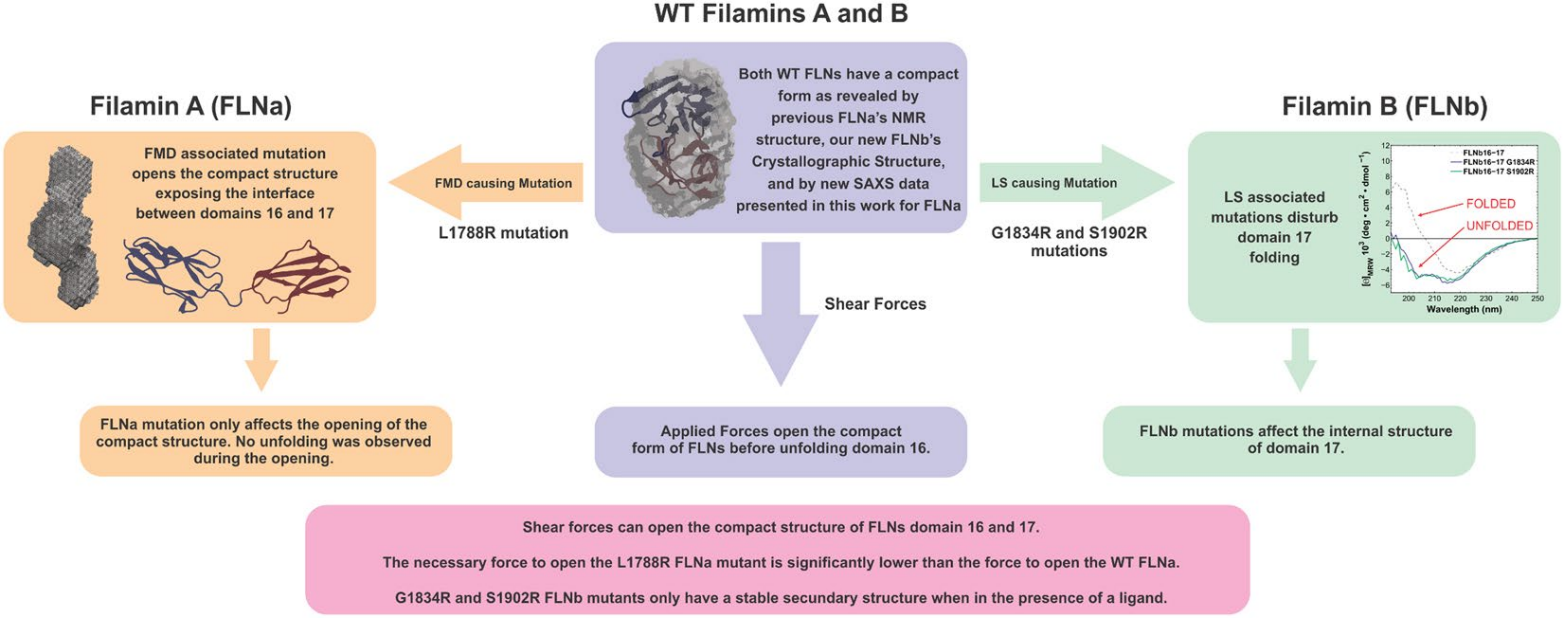
Schematic representation of effects of Skeletal Dysplasia mutations on FLN. In summary our results reveal that shear forces break a tight interaction between domains 16 and 17. Dysplasia associated mutations affect the structure of domains 16-17 either by destabilizing the internal structure of domain 17 or transforming 16-17 fragment from compact to an elongated form.

In summary, we presented here an investigation on the molecular mechanism of FMD and LS associated mutations on FLNs (see Fig. 7). First, the atomic structure of FLNb16-17 was resolved, revealing a very similar structure to FLNa16-17. Changes in folding due to three different mutations (1 in FLNa and 2 in FLNb) were investigated using SAXS, CD- and NMR spectroscopy and fluorometric thermal denaturation assays. SMD simulations were employed to investigate the mechanostability of both FLNa16-17 and FLNb16-17 in their WT and mutant forms. Dynamical network analysis was used to investigate the SMD trajectories, revealing amino-acid residues that play an important role in force propagation through the protein complex. Combined, our results do not provide direct molecular mechanisms for FMD and LS; nonetheless they show that the compact form of domains 16 and 17 is prone to be regulated by shear force. We also show that the mutations associated to FMD and LS may affect the mechanosensory functions of these domains. Dynamical network analysis revealed a smaller contribution of the mutation sites of FLNb compared to FLNa, which can be certainly explained by the results presented in Figs 3, 4 and 5; while the FLNa mutations affects the compact shape of the complex, from a compact to an elongated form, exposing the interface to the solvent and ligands, the FLNb mutations affect the internal structure of domain 17.

## Methods

### Recombinant proteins

Domains 16, 17 and domain pairs 16–17 of both FLNa and FLNb isoforms were PCR amplified from Human Microvascular Endothelial Cell λ cDNA library (Stratagene) according to the stable domain boundaries in FLNa16-17 structure (PDB ID: 2K7P)^22^ and cloned to pGTvL1 vector (Structural Genomics Consortium, University of Oxford) according to the ligation-independent cloning method^51^. Mutations were generated using the QuikChange Multi site-directed mutagenesis kit (Agilent Technologies). All final products were verified by sequencing. The Glutathione *S*-transferase (GST) fusion proteins were expressed in *Escherichia coli* BL21 Gold cells at 20–37 °C for 4–20 h. The cells were lysed using French Pressure Cell press (Thermo Fisher Scientific) or EmulsiFlex-C3 homogenizer (Avestin) and subsequently centrifuged at 48 000 g for 30 min. GST was cleaved by Tobacco Etch virus protease (Invitrogen, Life Technologies) at 4 °C for 16 h. Proteins cleaved from GST were eluted and further purified by size exclusion chromatography with a HiLoad 26/60 Superdex 75 column (GE Healthcare) in 20 mM Tris pH 8.0, 100 mM NaCl, 1 mM DTT using an ÄKTAprime chromatography system (GE Healthcare). Finally, the proteins were concentrated with Centriprep centrifugal filter units (Millipore). The homodispersity of the proteins was verified with analytical gel filtration and SDS-PAGE.

^15^N-labelled proteins for NMR experiments were expressed in *E.coli* in standard ^15^NH_4_Cl/M9 minimal medium. The proteins were purified in 50 mM sodium phosphate, 100 mM NaCl, 1 mM DTT using the same protocol as described above for the unlabeled fragments.

### Protein crystallography

Hanging drop vapor diffusion trials for FLNb16-17 were set up at room temperature using 1.6 mM protein mixed with equal volume of well solution in 200 nl drops. Mountable crystals were obtained in 3.5 M sodium formate pH 7.0. The crystals were transferred in well solution containing 20% glycerol and frozen in liquid nitrogen. The diffraction data were collected at ESRF beamline ID23–1 (wavelength 0.98 Å) at 100 K. The data were processed with XDS^52^ and solved with molecular replacement program Phaser^53^ using FLNa16-17 (PBD ID: 2K7P)^22^ as a search model. The model was built with ARP/wARP^54^ and Coot^55^ and refined with Refmac5^56^. The final stages of refinement were performed using the PBD-REDO server^57^. Processing and refinement statistics are summarized in Table 1. The Ramachandran plot shows 93% of all residues in the favored region and no more than 2 outliers. The structure factors and atomic coordinates were deposited in the PDB with ID 5DCP. All crystallographic figures were made with VMD^40^ and PyMol (version 1.7.2.1 Schrödinger, LCC).

**Table 1:** Data collection and refinement statistics for FLNb16-17 crystal structure.

	FLNb16-17^a^
**Data collection**	
Space group	P321
Cell dimensions	
*a*, *b*, *c* (Å)	80.62, 80.62, 118.02
α, β, γ (°)	90, 90, 120
Resolution (Å)	118–2.49 (2.55–2.49)^b^
*R*_sym_	11.1 (66.1)
*I*/*σI*	12.8 (3.1)
Completeness (%)	99.7 (96.2)
Redundancy	12.4 (6.9)
**Refinement**	
Resolution (Å)	12.4 (6.9)
No. reflections	15 238/802
*R*_work_/*R*_free_ (%)	19.6/23.8
No. atoms	
Protein	2389
Ligand/ion	0
Water	11
*B*-factors (Å^2^)	
Protein	65.9
Ligand/ion	0
Water	53.8
R.m.s. deviations	
Bond lengths (Å)	0.01
Bond angles (°)	1.62

^a^Diffraction data from a single crystal were used.

^b^Values in parentheses are for highest-resolution shell.

### NMR experiments

NMR samples were prepared in 50 mM NaH_2_PO_4_, 100 mM KCl, 1 mM DTT buffer at pH 6.8. D_2_O was added to obtain 10% solutions. Protein concentrations were 0.07–0.3 mM. All NMR spectra were collected using a Bruker Avance III HD 800 MHz NMR spectrometer, equipped with cryogenically cooled TCI ^1^H, ^13^C, ^15^N triple resonance probehead. Data were collected at 25 °C. Peptide binding assays were performed by stepwise addition of the GPIbα peptide to protein sample, resulting in approximate concentration ratios of (protein):(peptide), 1:0.5 and 1:1. At each step, the ^1^H ^15^N HSQC spectrum was measured for the detection of backbone amide group chemical shift preturbations. All spectra were processed with Topspin 3.5 and analyzed with NMRFAM-SPARKY 1.4^58^.

### SMD simulations

The atomic coordinates for FLNa16-17 were taken from the NMR-structure (PBD ID: 2K7P)^22^, whereas those for FLNb16-17 were taken from the chain A of the X-ray structure solved here (PBD ID: 5DCP). The L1788R mutated FLNa16-17, G1834R and S1902R mutated FLNb16-17 were generated by mutating the corresponding amino acids *in silico* in FLNa16-17 and FLNb16-17 atomic detailed structures, respectively, following the protocols described in QwikMD^41^. Systems were then solvated and the net charge of the protein was neutralized using sodium atoms as counter-ions, which were randomly arranged in the solvent. The CHARMM36 force field^59, 60^ along with the TIP3 water model^61^ was used to describe all systems. Before SMD simulations, the systems were first submitted to an energy minimization protocol for 1,000 steps and equilibrated using standard MD simulations. The MD simulations were performed employing the NAMD molecular dynamics package^39, 62^. The simulations were done assuming periodic boundary conditions in the NpT ensemble with temperature maintained at 300 K using Langevin dynamics for temperature coupling and kept at1 bar. A distance cut-off of 11.0 Å was applied to short range, non-bonded interactions, whereas long-range electrostatic interactions were treated using the particle-mesh Ewald (PME)^63^ method. The equations of motion were integrated using multiple time step scheme to update the van der Waals interactions every two steps and electrostatic interactions every four steps. The time step of integration was chosen to be 2 fs for all simulations performed. The systems were first equilibrated so that Cα-atoms of backbone were harmonically restrained either for 1 ns (WT fragments) or 50 ns (mutated fragments). Then the whole system was equilibrated freely for 1 ns (WT fragments) or 50 ns (mutated fragments).

SMD simulations^38^ were performed using constant velocity stretching (SMD-CV protocol) employing pulling speeds: 2.5 Å/ns. In all simulations, SMD was employed by restraining the position of N-terminus of domain 16 harmonically and the Cα-atom of the most C-terminal residue was assigned as SMD-atom. The force applied to the harmonic spring is then monitored during the time of the molecular dynamics simulation. The pulling point was moved with constant velocity along the z-axis and due to the single anchoring point and the single pulling point the system is quickly aligned along the z-axis. SMD simulations were repeated and comparable results were obtained. All analyses of MD trajectories were carried out employing VMD^40^ and its plugins. Force propagation networks were analyzed using same protocol described in ref. 44.

### SAXS

SAXS measurements were performed at ESRF (European Synchrotron Radiation Facility) beamline BM29^64^ equipped with PILATUS 1 M image plate. Sample to detector distance of 2.9 m and wavelength of 0.1 nm (momentum transfer range 0.01 < q < 5 nm−1) were used. The data were collected at +20 °C. All measurements were done in three different protein concentrations (1.0, 3.0 and 5.0 mg/ml). DTT was added to the gel filtration buffer to have final concentration of 10 mM. The data processing was conducted using the standard procedures in the ATSAS software package^65^. The data reduction was conducted with PRIMUS^66^. Distance distribution functions were calculated with DATGNOM^67^ and Porod volumes with program DATPOROD^67^. *Ab initio* shape envelopes were created by running ten rounds of GASBOR^68^ which were then aligned, averaged and filtered by DAMAVER^69^. The averaged models were finally refined by DAMMIN^70^ against the scattering data. SUPCOMB^71^ was used to overlay the high-resolution structure and the ab initio shape envelope with minimal normalized spatial discrepancy. Radius of gyration and maximum dimensions were calculated from Guinier analysis and distance distribution functions, respectively. All measured fragments behaved well in solution and Guinier analysis indicated no apparent particle aggregation or repulsion (Supplementary Table S1). The scattering data along with the *ab initio* shape envelopes were deposited in the SASBDB (SASDB32 and SASDB42)^72^.

### CD spectroscopy

CD spectroscopy measurements were conducted with Jasco J715 spectropolarimeter at 20 °C using 10–30 µM protein in 10 mM potassium phosphate, 100 mM (NH_4_)_2_SO_4_, pH 8.0. Spectra were recorded for 190–290 nm with 20 nm/min scanning rate in a cuvette with 0.5 mm path length. GP1bα-peptide was used in equimolar concentrations with the WT, G1834R and S1902R FLNb17 fragments when recording the data in the presence of the peptide. The peptide was incubated with the proteins for 1 h on ice prior to measurements. The data were processed using MATLAB R2014a (The MathWorks, Inc.).

### Fluorescence-based thermal stability assays

Thermal stabilities of the WT and mutated FLN fragments were determined using Bio-Rad C1000 Thermal Cycler, CFx96 Real-Time system (Bio-Rad Laboratories) with SYPRO Orange fluorescent dye (Invitrogen)^73^. A temperature gradient from 20 to 95 °C was used with 0.5 °C increment every 30 seconds. Samples contained 100–200 μM protein and 5 X SYPRO Orange dye in total volume of 25 μl. The data was collected from three individual experiments and plotted using MATLAB R2014a (The MathWorks, Inc.).

### Limited proteolysis

The WT and G1834R and S1902R mutated FLN16–17 were studied by limited proteolysis assay using α-chymotrypsin (Sigma) in a ratio of 1:1000 (wt/wt). The proteolysis occurred at room temperature in 20 mM Tris pH 8.0, 100 mM NaCl, 1 mM DTT. Samples collected from various incubation intervals were separated according their molecular mass using 12% gels in SDS-PAGE. The entire experiment was repeated twice and reproducible degradation patterns were obtained.

### Binding assays

The GPIbα-peptide (^599^LRGSLPTFRSSLFLWVRPNGRV^622^, UNIPROT ID P07359) purchased from GenScript was coupled to NHS-activated Sepharose TM 4 Fast Flow (GE Healthcare) following the manufacturer’s instructions. Concentration series (0, 5, 10, 25, 50, 100 and 200 μM) of WT and G1834R and S1902R mutated FLNb16-17 fragments were prepared in binding buffer (20 mM Tris; pH 7.4, 150 mM NaCl, 1% Triton X-100). 50 µl of Sepharose was incubated with the samples for 1 h at room temperature. The Sepharose was then washed three times with 500 µl of binding buffer using a centrifuge at 2000 × g for 2 min. The proteins were eluted with 20 µl of 2x SDS-electrophoresis sample buffer and separated according to the molecular mass using 12% gels in SDS-PAGE. FLN binding to the GPIbα-peptide was quantified by protein staining and expressed as FLN binding the GPIbα-peptide, and normalized to maximal FLN binding in each experiment. Experiments for each FLN fragment were repeated three times. ImageJ was used for measuring the intensities of the Coomassie-stained protein bands and GraphPad Prism 4 (GraphPad Software) was used for plotting the data with standard error of the mean of each FLN fragment.

### Sequence alignments

Sequence comparisons of vertebrate FLNa and FLNb protein sequences, retrieved from Uniprot^74^, were performed for sequences that can be identified with protein names (filamin-a/filamin-b), have more than 2,000 residues, and are available for both FLNa and FLNb. The resulting 19 sequences for both FLNs were aligned with Malign^75^ in Bodil^76^ by using Structure-based matrix^77^ with gap penalty of 90. The high gap penalty ensures that in the case of inserted/deleted domains, the gaps are not spread between nearby domains. The Supplementary Figure 2 was prepared with Alscript-2.07a^78^.

## Electronic supplementary material

Supplementary material
