# Supplementary material for: Skeletal Dysplasia Mutations Effect on Human Filamins’ Structure and Mechanosensing
Source: Sci Rep. 2017 Jun 26;7:4218. doi: 10.1038/s41598-017-04441-x (PMC5484675; doi:10.1038/s41598-017-04441-x)
Supplement: Supplementary file 1 — Supplementary material [file 41598_2017_4441_MOESM1_ESM.pdf]

Skeletal Dysplasia Mutations Effect on Human Filamins' Structure and Mechanosensing

Jonne Seppälä[1], Rafael C. Bernardi[2], Tatu Haataja[1], Maarit Hellman[4], Olli T. Pentikäinen[1], Klaus Schulten[2,3#], Perttu Permi[1,4], Jari Ylänne[1] & Ulla Pentikäinen[1]*

[1] Department of Biological and Environmental Science and Nanoscience Center, University of Jyvaskyla, P.O Box 35, Survontie 9 C, FI-40014 Jyvaskyla, Finland
[2] Beckman Institute for Advanced Science and Technology, University of Illinois at Urbana-Champaign, 61801, USA.
[3] Department of Physics, University of Illinois at Urbana-Champaign, 61801, USA.
[4] Department of Chemistry, University of Jyvaskyla, P.O Box 35, Survontie 9 C, FI-40014 Jyvaskyla, Finland

*corresponding author: email: ulla.m.pentikainen@jyu.fi, tel: +358408053916, fax: +358 14 617 239

in memoriam

Supporting Information

Table S1. SAXS derived structural parameters for FLNa16–17 WT and L1788R mutant

Sample	*c (mg/ml)*	R_g *(nm)*[a]	D_{max} *(nm)*[b]	V_p *(nm³)*[c]	M_w *(kDa)*[d]
FLNa16–17		**1.8**	**6.2**	**30.7**	**18.1**
	1.0	1.8	6.4	31.0	18.2
	3.0	1.8	6.1	32.7	19.2
	5.0	1.7	5.2	29.9	17.6
FLNa16–17 L1788R		**2.4**	**8.3**	**36.8**	**21.6**
	1.0	2.4	8.4	35.8	21.1
	3.0	2.4	8.5	37.8	22.2
	5.0	2.4	8.4	37.8	22.2

The values in bold are calculated for the merged scattering of high and low concentration data.
[a] Estimated from Guinier analysis in PRIMUS [66]. Estimated error is ±0.1 nm.
[b] Calculated with DATGNOM [67]. Estimated error is ±0.5 nm.
[c] Calculated with DATPOROD [67] using the regularized scattering from *P(r)* analysis.
[d] Calculated from V_p by dividing the volume with 1.7 [65]. Calculated M_w from sequence are 18.7 kDa for FLNa16-17.

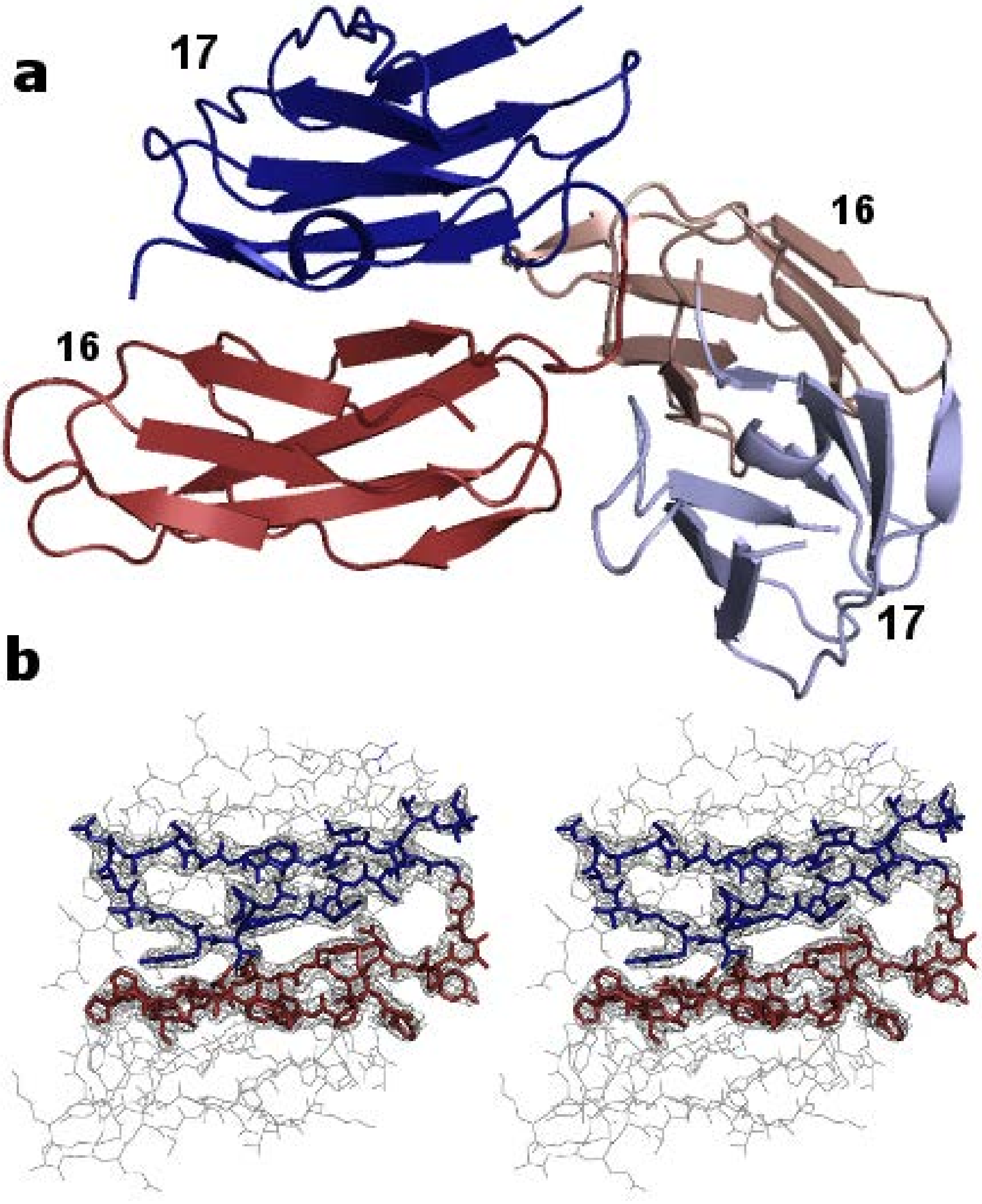

Supplementary Figure 1. The asymmetric unit of FLNb16-17 crystal.

a. The asymmetric unit of FLNb16–17 crystal**. b.** A stereo-view of FLNb16-17. An electron density map (F_o –F_c) rendered at σ level 1.5 calculated from the refined model is shown for domain-domain interface.

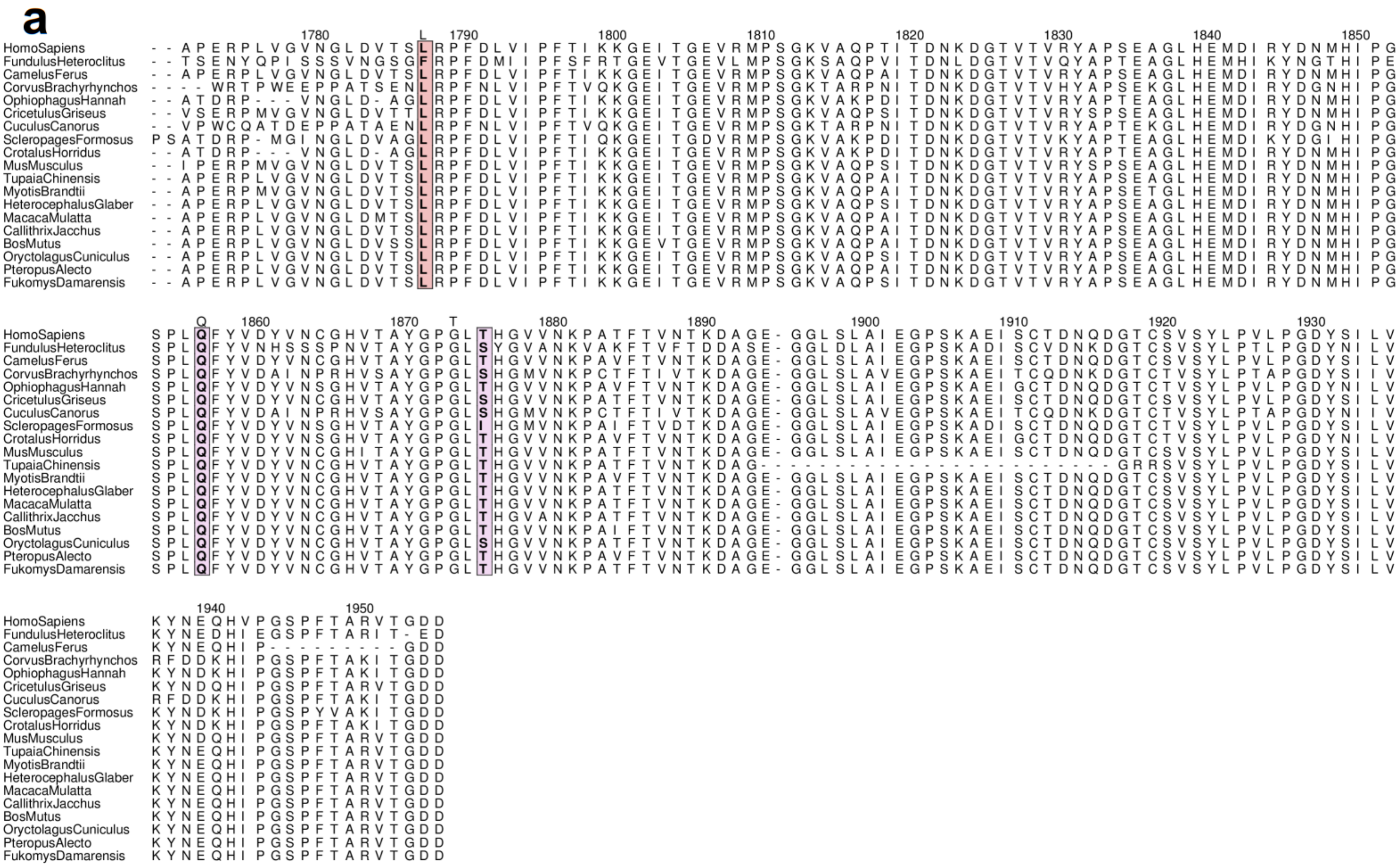
a
HomoSapiens
FundulusHeteroclitus
CamelusFerus
CorvusBrachyrhynchos
OphiophagusHannah
CricetulusGriseus
CuculusCanorus
ScleropagesFormosus
CrotalusHorridus
MusMusculus
TupaiaChinensis
MyotisBrandtii
HeterocephalusGlaber
MacacaMulatta
CallithrixJacchus
BosMutus
OryctolagusCuniculus
PteropusAlecto
FukomysDamarensis
1780
1790
1800
1810
1820
1830
1840
1850
1860
1870
1880
1890
1900
1910
1920
1930
1940
1950

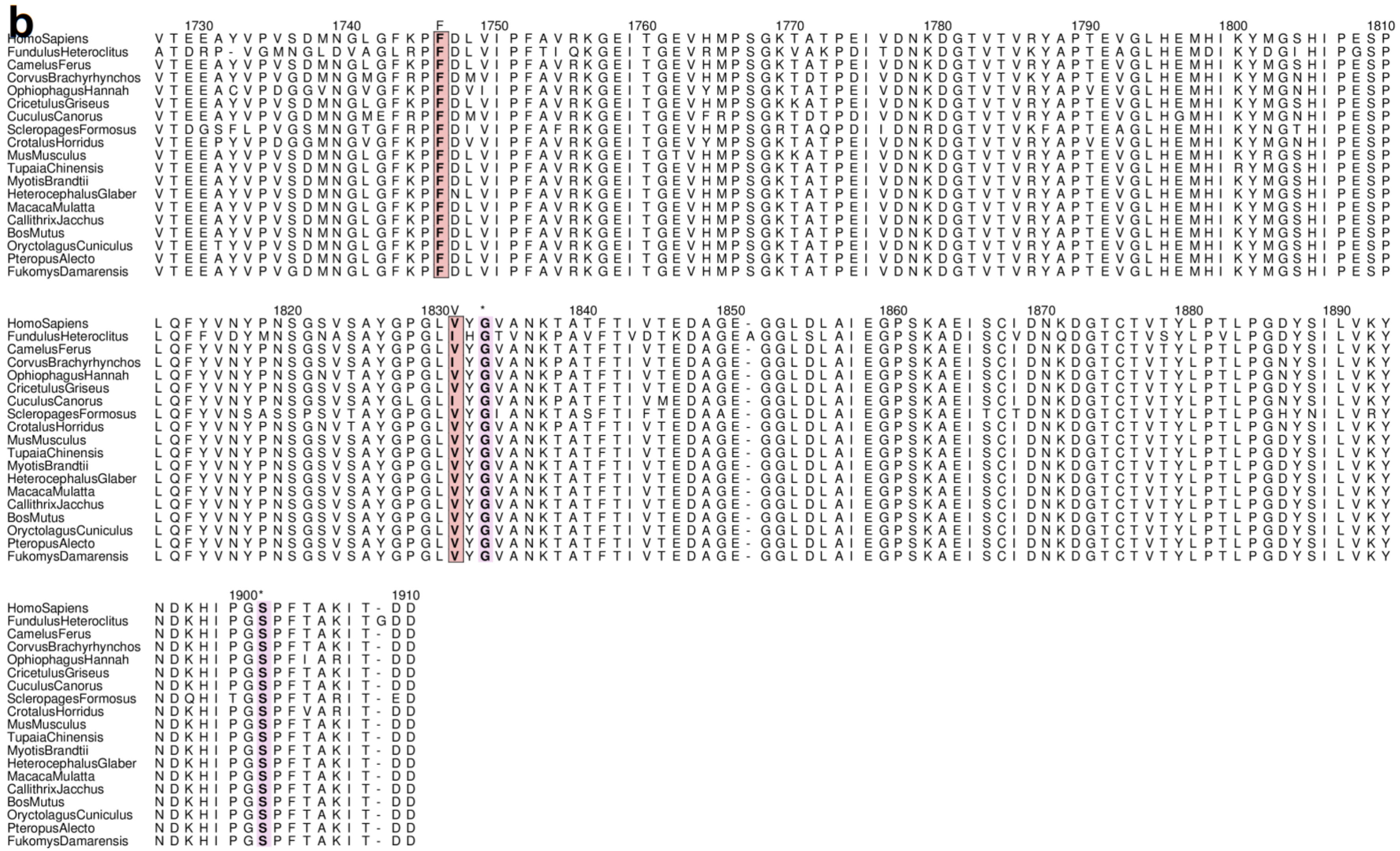

Supplementary Figure 2. The sequence aligment of vertebrate FLNs.

a. The sequence aligment of vertebrate FLNa sequences shows that L1788 whose mutation to arginine causes FMD is conserved among vertebrate FLNas. Q1857 and T1826, the residues, which show the strongest correlation between the internal motion of domains 16 and 17, are also conserved either explicitly (Q1857) or alternating with structural analogue (T1826 with serine). **b.** The sequence aligment of vertebrate FLNb sequences shows that G1834 and S1902 whose mutation to arginine causes LS are conserved among vertebrate FLNbs. F1747 and V1832, the residues, which show the strongest correlation between the internal motion of domains 16 and 17, are also conserved either explicitly (F1747) or alternating with structural analogue (V1832 with isoleusine).

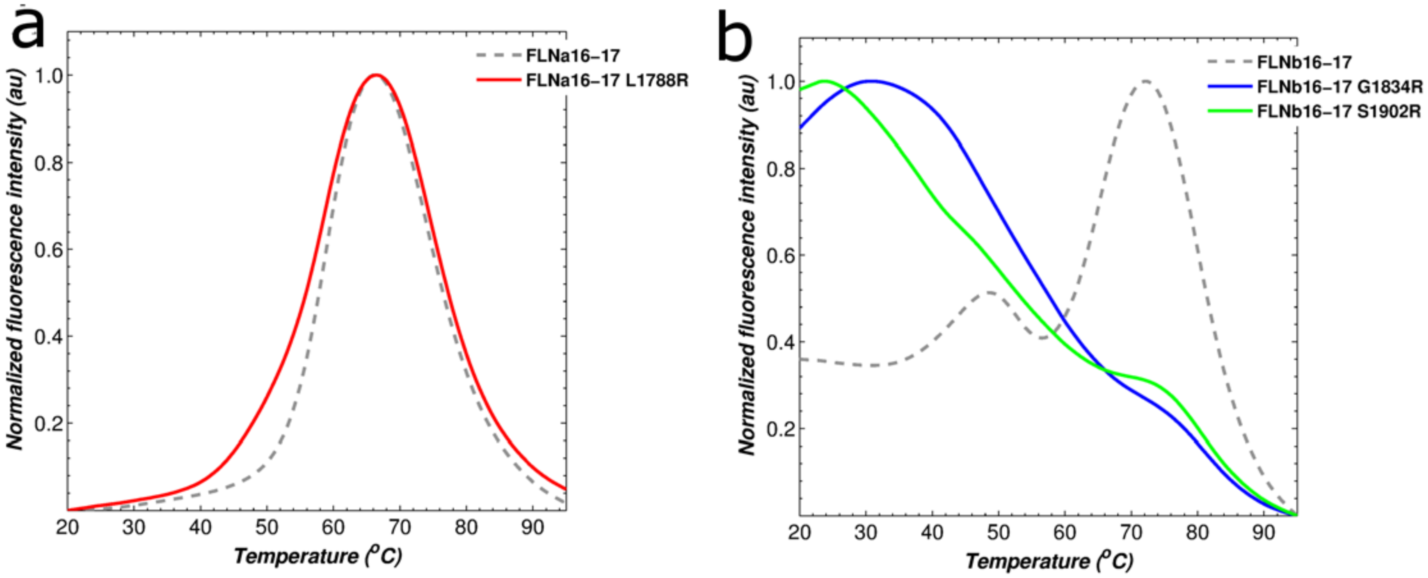

Supplementary Figure 3 - The effects of skeletal dysplasia associated mutations on thermal stabilities of FLN fragments.

a. The fluorescence-based thermal stability assays of WT and L1788R mutated FLNa16-17 show that L1788R mutation does not have significant effect on the thermal stability of FLNa16-17 fragment since both WT and L1788R mutated fragments have very similar distinct dye binding curves. **b.** The fluorescence-based thermal stability assays of the G1834R and S1902R mutated FLNb16–17 fragments have nearly featureless decaying curves, as is typical for un- or misfolded proteins whereas as WT fragment shows a distinct dye binding curve.

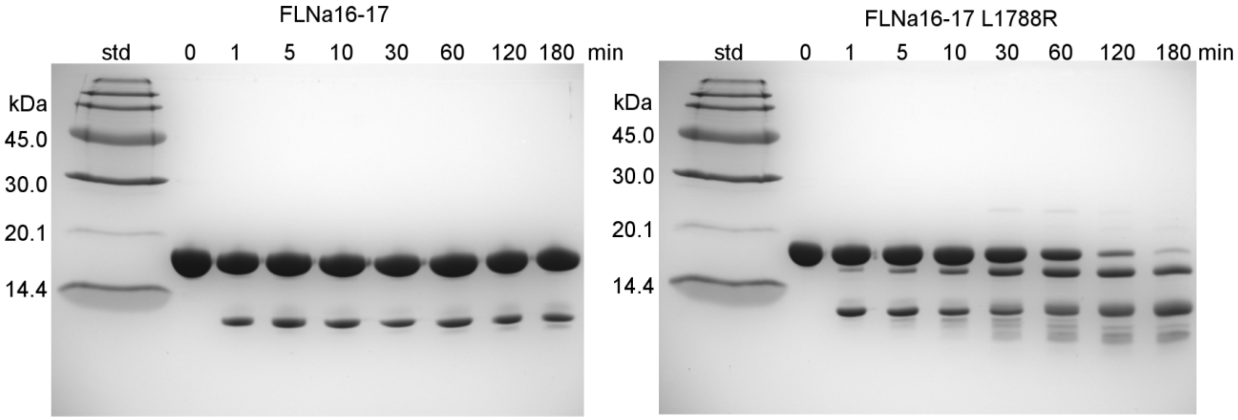

Supplementary Figure 4. FMD-linked L1788R mutation makes FLNa16-17 more prone to proteolytic digestion.

α-Chymotrypsin digestion of the 20 kDa WT and L1788R mutated FLNa16–17 fragments after 0, 1, 5, 10, 30, 60 120 and 180 min incubation. Molecular weight standard (std) is shown in the left panel in both gels. The α-chymotrypsin treatment indicates that the mutated fragments are more prone to proteolytic activity than the WT proteins.

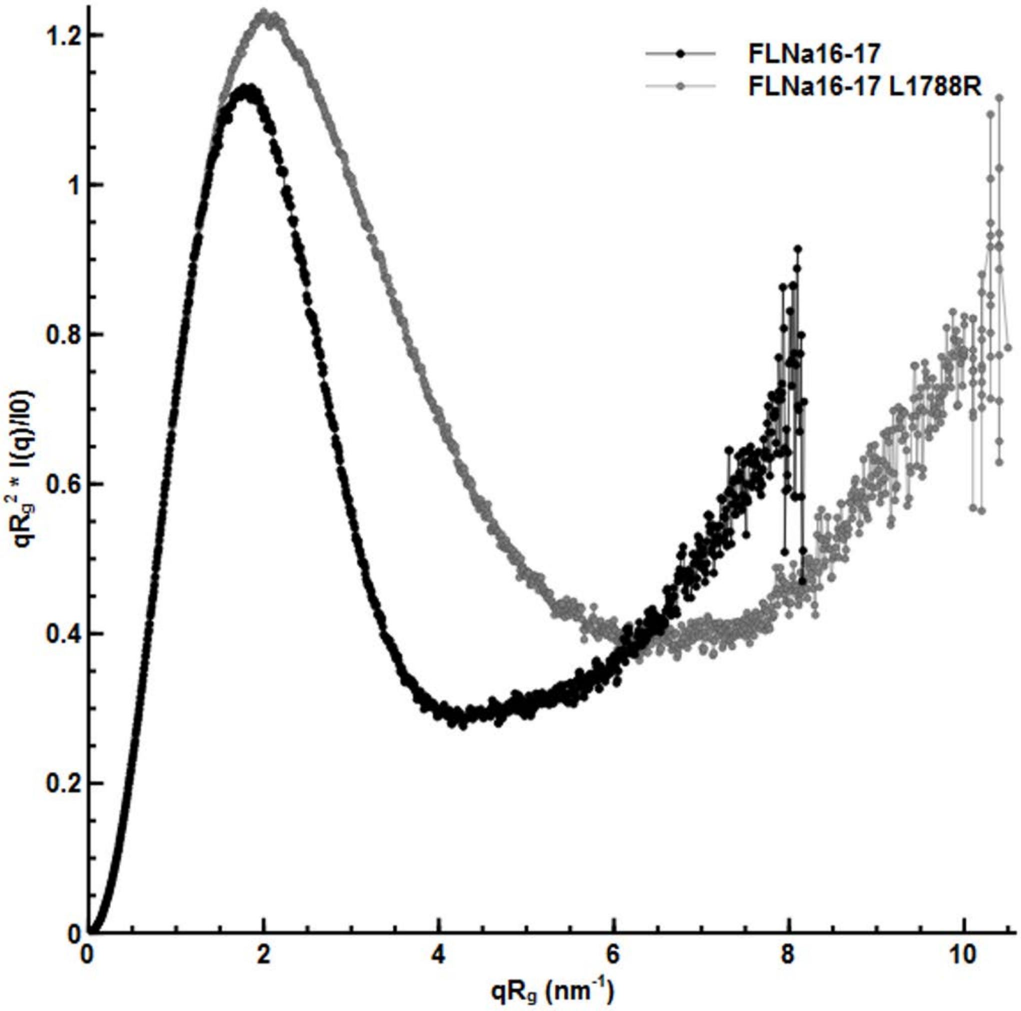

Supplementary Figure 5. FMD-linked L1788R mutation makes FLNa16-17 more flexible compared to WT.

Normalized Kratky plots [38] for WT and L1788R mutated FLNa16–17 obtained from SAXS measurements show that L1788R mutated fragment is more flexible than the WT fragment.

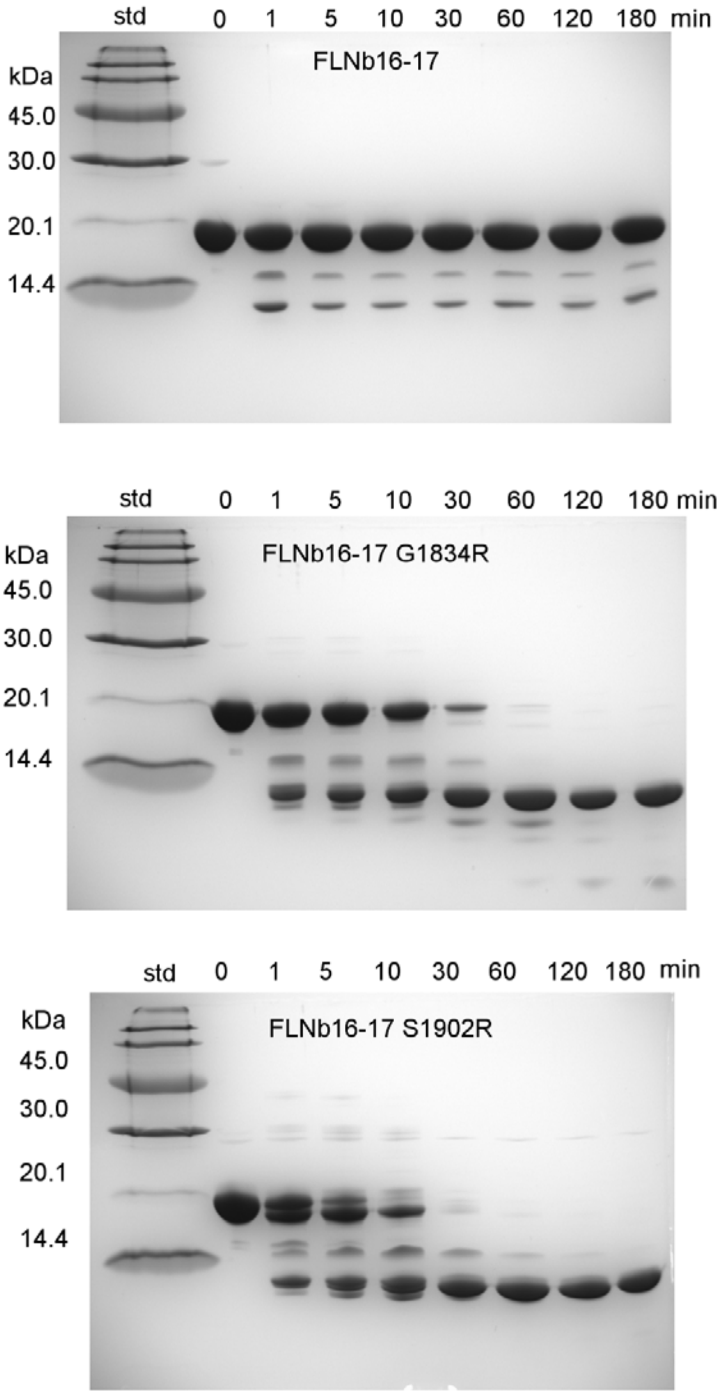

Supplementary Figure 6. FMD-linked G1834R and S1902R mutations make FLNb16-17 more prone to proteolytic digestion.

α-Chymotrypsin digestion of the 20 kDa WT, G1834R and S1902R mutated FLNb16–17 fragments after 0, 1, 5, 10, 30, 60 120 and 180 min incubation. Molecular weight standard (std) is shown in the left panel in all gels. The α-chymotrypsin treatment indicates that the mutated fragments are more prone to proteolytic activity than the WT proteins.

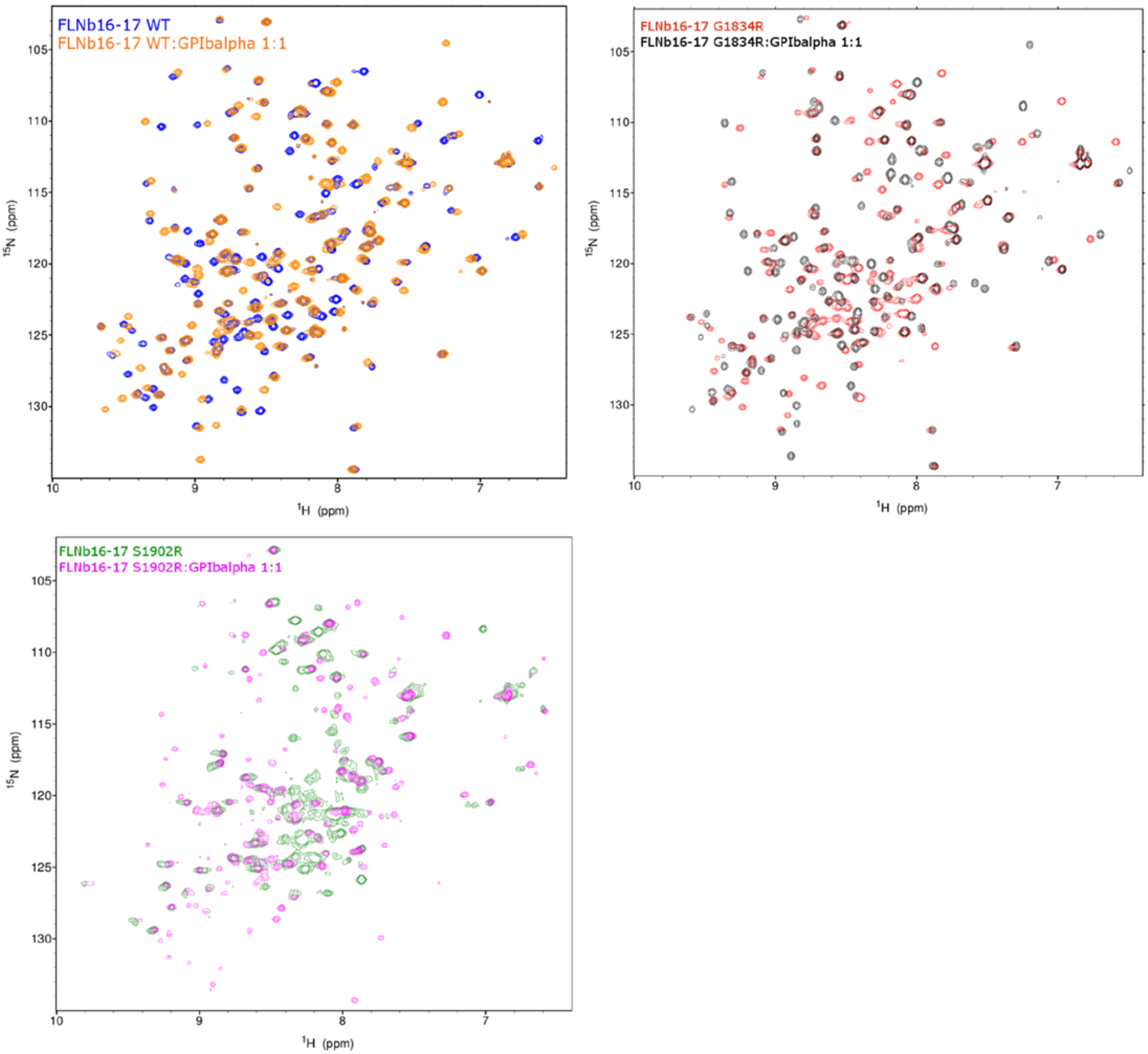

Supplementary Figure 7. The overlay of ^{1}H ^{15}N HSQC spectra of FLNb16-17 WT, G1834R, and S1902R collected before and after titration with GP1bα-peptide.

From the comparison of the spectra before and after titration with GPIbα-peptide (prot:pep=1:1) can be seen that while the binding of the peptide to G1834R mutant is similar than to WT, the peptide binding to S1902R mutated fragment induces the formation of more ordered structure.

.
